# Novel Orthogonally Hydrocarbon-Modified Cell-Penetrating Peptide Nanoparticles Mediate Efficient Delivery of Splice-Switching Antisense Oligonucleotides In Vitro and In Vivo

**DOI:** 10.3390/biomedicines9081046

**Published:** 2021-08-19

**Authors:** Safa Bazaz, Tõnis Lehto, Rahel Tops, Olof Gissberg, Dhanu Gupta, Burcu Bestas, Jeremy Bost, Oscar P. B. Wiklander, Helena Sork, Eman M. Zaghloul, Doste R. Mamand, Mattias Hällbrink, Rannar Sillard, Osama Saher, Kariem Ezzat, C. I. Edvard Smith, Samir EL Andaloussi, Taavi Lehto

**Affiliations:** 1 Clinical Research Center, Department of Laboratory Medicine, Karolinska Institutet, Karolinska University Hospital Huddinge, 14186 Huddinge, Sweden; safa.bazaz.hidush@ki.se (S.B.); ogolle@gmail.com (O.G.); dhanu.gupta@ki.se (D.G.); bbestas@gmail.com (B.B.); jeremy.bost@ki.se (J.B.); oscar.wiklander@ki.se (O.P.B.W.); eman_zagloul@hotmail.com (E.M.Z.); doste.mamand@ki.se (D.R.M.); mattias.hallbrink@ki.se (M.H.); osama.saher@ki.se (O.S.); kariem.ezzat.ahmed@ki.se (K.E.); edvard.smith@ki.se (C.I.E.S.); samir.el-andaloussi@ki.se (S.E.A.); 2 Department of General Biology, Cihan University-Erbil, Kurdistan Region, Erbil 44001, Iraq; 3 Institute of Technology, University of Tartu, Nooruse 1, 50411 Tartu, Estonia; tonis.lehto@ut.ee (T.L.); rahel.tops@ut.ee (R.T.); helena.sork@ut.ee (H.S.); 4 Department of Pharmaceutics, Faculty of Pharmacy, Alexandria University, El-Khartoum Square, Azareeta, Alexandria 21521, Egypt; 5 Department of Biomedical Sciences, Cihan University-Erbil, Erbil 44001, Kurdistan Region, Iraq; 6 PeptiSystems AB, Virdings Allé 22, 75450 Uppsala, Sweden; rannar.sillard@gmail.com; 7 Department of Pharmaceutics and Industrial Pharmacy, Faculty of Pharmacy, Cairo University, Cairo 11562, Egypt

**Keywords:** cell-penetrating peptides, splice-switching oligonucleotides, oligonucleotide delivery, cellular uptake, endocytosis

## Abstract

Splice-switching therapy with splice-switching oligonucleotides (SSOs) has recently proven to be a clinically applicable strategy for the treatment of several mis-splice disorders. Despite this, wider application of SSOs is severely limited by the inherently poor bioavailability of SSO-based therapeutic compounds. Cell-penetrating peptides (CPPs) are a class of drug delivery systems (DDSs) that have recently gained considerable attention for improving the uptake of various oligonucleotide (ON)-based compounds, including SSOs. One strategy that has been successfully applied to develop effective CPP vectors is the introduction of various lipid modifications into the peptide. Here, we repurpose hydrocarbon-modified amino acids used in peptide stapling for the orthogonal introduction of hydrophobic modifications into the CPP structure during peptide synthesis. Our data show that α,α-disubstituted alkenyl-alanines can be successfully utilized to introduce hydrophobic modifications into CPPs to improve their ability to formulate SSOs into nanoparticles (NPs), and to mediate high delivery efficacy and tolerability both in vitro and in vivo. Conclusively, our results offer a new flexible approach for the sequence-specific introduction of hydrophobicity into the structure of CPPs and for improving their delivery properties.

## 1. Introduction

Splice-switching therapy with splice-switching oligonucleotides (SSOs) has recently gained significant momentum for the treatment of various mis-splice disorders [1]. Treatments with SSOs usually lead to the restoration of correct splicing, production of a novel splice-variant or directing from one splice-variant to another. Recent successful clinical trials for neuromuscular disorders, such as for Duchenne muscular dystrophy (DMD) and spinal muscular atrophy (SMA), have demonstrated the excellent potential that SSO therapeutics can offer for the treatment of human disease. Despite these major breakthroughs, issues with the efficacy of SSO therapies persists and this is primarily attributed to the limited bioavailability of SSOs in the target tissues/cells. In fact, it has been estimated that as low as 0.1% of the total administered SSO dose is ultimately available at their target site for engagement, while the majority of material is lost due to limited tissue/cellular uptake, clearance by liver and excretion by kidneys, but also caused by unspecific binding to proteins in both extra- and intracellular compartments [2]. Therefore, there is clearly enormous room for improving the delivery and efficacy of SSO-based therapeutic modalities.

One way to improve the delivery and bioavailability of SSOs in tissues/cells is by utilizing various drug delivery systems (DDSs). A group of DDSs that has lately gained increasing attention are short delivery peptides called cell-penetrating peptides (CPPs) [3]. CPPs are cationic and/or amphipathic peptides up to 30 amino acids in length [4] that have been successfully applied for the delivery of a wide variety of nucleic acid-based molecules, ranging from short oligonucleotides (ONs) to larger plasmid molecules [5]. CPPs can be used to formulate SSOs by two main strategies, either via covalent conjugation or by formation of discrete nanoparticles. Compared to other delivery vectors, CPPs are known for their high cellular permeability and ability to enter a broad range of cell types and are considered to have a favorable toxicity and immunological safety profile compared to other synthetic vectors, such as cationic lipids or polymers [6]. CPPs are known to enter cells by using various energy-dependent endocytic mechanisms, while there are also reports on the direct translocation mechanism for cellular entry [3].

The hydrophobic nature of the CPPs, as well as the inclusion of basic amino acids, have been reported to play a vital role in the cellular uptake and the subsequent release from endosomes [7]. Over the years, many attempts have been made to enhance the delivery properties of CPPs or CPP-nucleic acid complexes. These include various strategies which aim to improve the structural features of the CPP sequences. For example, fine-tuning of their secondary structure, especially the α-helical motif, has been shown to have a significant impact on protein-protein interactions and cellular internalization of some of the CPPs [8]. To this end, several approaches have been developed to stabilize the α-helical secondary structure in peptides via side-chain crosslinking or peptide stapling in order to improve their cellular uptake and stability to proteases [9]. One such approach is ruthenium-catalyzed ring-closing metathesis (RCM), where α,α-disubstituted amino acids modified with hydrocarbon (alkenyl) side-chains of varying lengths are introduced to the peptide backbone and the α-helical structure locked by covalently joining the alkenyl side-chains after one or two helical turns [10].

Another commonly used approach for improving their delivery efficacy is the N-terminal modification of CPPs with fatty acids to increase their hydrophobicity. For example, increased hydrophobicity by stearylation has been shown to increase the stability of CPP/nucleic acid complexes [11], enhance the association of complexes with the cell membrane [11,12] and increase the cellular uptake and endosomal escape [13]. On the other hand, N-terminal modification of CPPs with fatty acids can be a double-edged sword, as it also increases their membrane activity and hemolytic activity [12], which can lead to possible adverse effects. Hence, we propose a strategy where shorter hydrophobic modifications are distributed evenly over the peptide sequence to yield the same net hydrophobicity as longer fatty acids would. 

To this end, we have recently developed several amphipathic peptide analogs based on a sequence called hPep1. In this study, we repurpose the hydrophobic alkenyl-alanines used in RCM to orthogonally introduce hydrophobicity into hPep1 peptide in order to improve its ability to efficiently formulate and deliver SSOs. Here we evaluate the viability of this approach by varying the number and length of these hydrocarbon modifications for improving the in vitro and in vivo delivery of SSO therapeutics.

## 2. Methods and Materials

### 2.1. Cell-Penetrating Peptides

All peptides were ordered from Pepscan Presto (Lelystad, The Netherlands) with >90% purity. The peptides are C-terminally amidated and have a free amine in the N-terminus. The purities of the peptides were obtained by UPLC (Ultra Performance Liquid Chromatography) with a linear gradient system (gradient: 5–55% B in 2 min, flow: 1 mL/min, eluent A: 100% H_2_O + 0.05% TFA; eluent B: 100% ACN + 0.05% TFA) using an C18 RP-HPLC column and detection at 215 nm. All peptides were provided as white powders. The hydrophobicity of the peptides was evaluated by UPLC with the following ACN gradient: from 5 to 90% in 13 min, 90–100% in 1.5 min followed by 2.5 min at 100% ACN in a C18 column.

### 2.2. Splice-Switching Oligonucleotides

In cell culture studies we used SSO705 with or without 5′-AlexaFluor568 fluorescent label (Seq: 5′-ccucuuaccucaguuaca-3′, small letters are indicating phosphorothioate 2′-O-methyl modified RNA). Interleukin 6 Signal Transducer (IL6ST) SSO: 5′-accuuccacacgaguuguac-3′ was used as a negative control. For evaluating the in vivo biodistribution of peptide/oligonucleotide complexes, fluorescently labelled SSO targeting IL6ST was used (Seq: 5′-Cy5-ggTcugGaugGuccTa, small letters are indicating phosphorothioate 2′-O-methyl modified RNA and capital letters are indicating locked nucleic acid modified RNA). Oligonucleotides were synthesized by Sigma Aldrich (Tokyo, Japan).

### 2.3. Peptide/Oligonucleotide Complex Formulation

Peptide/SSO complexes were prepared in HEPES-buffered glucose (HBG) (20 mM HEPES, pH = 7.4, 5% glucose) at different peptide-to-SSO molar ratios (MRs) 1:1 to 10:1. For that, appropriate amounts of peptide (100 µM) and SSO (10 µM) working solutions where first diluted to an equal volume with HBG and then pipetted together and left for complex formation at RT for 30 min. 

### 2.4. Complex Stability Assay

Peptide/SSO complexes were formed at MR5 using three different peptides: hPep1, hPep2, and hPep3. The complexes were then transferred to black 96-well plates containing SYBR™ Gold dye (Invitrogen™, Waltham, MA, USA) to measure the amount of accessible SSO in the complexes with SynergyMX fluorometer (BioTek, Winooski, VT, USA). For complex stability evaluation, competitive anion binder hexametaphosphate (HMP) was added at different concentrations from 0.04 to 40 µg/mL and the amount of released oligonucleotide was measured after the HMP addition. Measurements were done in duplicates and results show the mean of three independent experiments (mean ± SEM, *n* = 3).

### 2.5. Cell Cultures

HeLa pLuc705 reporter cells, first developed by Prof R. Kole and colleagues [14], were cultivated in DMEM (Dulbecco’s Modified Eagle’s Medium; Thermo Fisher Scientific, Waltham, MA, USA) with 10% FBS (fetal bovine serum) with 1% (Penicillin: 100 U/mL, Streptomycin: 100 μg/mL) and maintained in a water-jacketed incubator at 37 °C and 5% CO_2_ atmosphere. 

### 2.6. Splice-Switching Assay

For the splice-switching activity measurements either 7000 or 35,000 cells were seeded 24 h prior to treatment into 24 or 96-well plates, respectively. Then complexes were added to the cells in 1/10 of the final volume of the cell media at 24 h post seeding. For varying the cell treatment, concentration complexes were diluted prior the treatment to appropriate concentration to retain the 1/10 of the final volume of the cell media during treatment. After 24 h of treatment, media was removed and cells were lysed in 0.1% Triton X-100 (Sigma-Aldrich) for 30 min at room temperature. The luciferase activity was measured from the lysates with the Luciferase Assay Kit (Promega, Madison, WI, USA) under GLOMAX 96 microplate luminometer (Promega) and normalized to the protein content (Bio-Rad Protein Assay Kit II, BioRad, Hercules, CA, USA). The data were further normalized to untreated cells and the splice switching activity was presented as a fold-increase over untreated cells.

For the chloroquine-induced endosomal release, the cells were treated at 200 nM hPep3/SSO complexes for 4 h, followed by 2 h challenging with 50 μM chloroquine. All stages included an intermediate washing step. Luciferase expression was measured after 18 h.

### 2.7. Quantitative Uptake

20,000 cells per well were seeded on 96-well plates 24 h prior to treatment. Cells were treated with peptide/AF568-SSO complexes (using peptides hPep1, hPep2, hPep3) with final concentrations of 25–100 nM at MR 5 in both serum-free and serum-containing conditions for 4 h. Complexes were formed in MQ water. After treatment, cells were washed two times with 100 µg/mL heparin (in DPBS). Cells were then lysed with 50 µL of 0.1% Triton X-100 per well and the fluorescence was measured at ex 568 nm, em 603 nm with SynergyMX fluorometer (BioTek) and normalized to the protein content (Bio-Rad Protein Assay Kit II, BioRad). The values represent the mean of at least three independent experiments done in duplicates (mean ± SEM).

### 2.8. Confocal Microscopy

20,000 HeLa pLuc705 cells/well were seeded in Nunc^®^ Lab-Tek II^®^ glass-bottom chamber slides (Thermo Scientific™, Waltham, MA, USA). After 24 h of adherence, hPep3/AF568-SSO complexes were added to cells at a 200nM final concentration of SSO in serum-containing media. After 4 h, the nuclei were stained with Hoechst 33342 (Thermo Scientific™) for 10 min, then media was removed and cells washed 3 times with PBS. Live-cell imaging was performed in a stage-top incubator at 37 °C with 5% CO_2_. Imaging was carried out using a confocal microscope (A1R confocal, Nikon, Tokyo, Japan) and analyzed by the NIS-Elements software (Nikon, Tokyo, Japan).

### 2.9. Nanoparticle Size Measurements

The size of the peptide/SSO complex was measured by Nanoparticle Tracking Analysis (NTA). The complexes were formulated as described above and measured on the NS500 instrument (Malvern Instruments, Malvern, Worcestershire, UK). The movement of nanoparticles for each sample was recorded five times (30 s each video). In order to process the videos, the camera gain and minimum detection threshold was set to 14 and 7, respectively.

### 2.10. Cytotoxicity Evaluation

Metabolic activity of the cells was observed by WST-1 assay (Roche, Clifton, NJ, USA) as instructed by the manufacturer’s protocol. HeLa pLuc705 cells were seeded in 96-well plates and treated with complexes for 24 h at 12.5–200 nM final SSO concentration. Thereafter, the media was changed, and WST-1 reagent was added for 4 h. The toxicity was evaluated by measuring the absorbance of the formazan product at 450 nm and the background at 650 nm on Spectra Max (Molecular Devices, Silicon Valley, CA, USA). The results were normalized to untreated cells (UT).

### 2.11. RNA Expression Analysis of Splice-Switching

The corrected percentage of luciferase mRNA was determined by total RNA isolation from the cells using the standard phenol-chloroform extraction protocol with Trizol (Thermo Fisher Scientific, Waltham, MA, USA). A high-capacity cDNA reverse transcription kit was used to perform each RT-PCR reaction with 500 ng of isolated RNA (Applied Biosystems, Waltham, MA, USA). The total volume was 20 µL and the primers had a following sequence: Fwd-5′-TTGATATGTGGATTTCGAGTCGTC; Rev-5′-TGTCAATCAGAGTGCTTTTGGCG. The RT-PCR protocol was as follows: 55 °C for 35 min, followed by 15 min at 95 °C for reverse transcription. Secondary nested PCR was performed using two microliters of the RT-PCR using Hot Taq plus DNA polymerase (Qiagen, Germantown, MD, USA). The PCR program was performed for 30 cycles (94 °C for 30 s, then 55 °C for 30 s, then 72 °C for 30 s, and lastly 72 °C for 10 min). A 2% agarose gel for the PCR products was used in 1 × TAE buffer and detected by SYBR Gold staining (Invitrogen). The Versadoc imaging system provided with a CCD camera was used to analyze gels (BioRad, Hercules, CA, USA). Quantity One software was used to analyze band intensities (BioRad, Hercules, CA, USA). The percentage of splice-correction was expressed as [Band intensity of corrected RNA × 100/(The sum of corrected and uncorrected RNA band intensities)].

### 2.12. Endocytosis Inhibition Assay

50,000 cells per well were seeded on 24-well plates in serum-containing DMEM 24 h prior to treating them for 30 min with the following inhibitors: 4 µM cytochalasin D for macropinocytosis inhibition; 15 µM chlorpromazine for clathrin-mediated endocytosis inhibition; 12.5 µM nystatin for caveolae-mediated endocytosis inhibition; 6 mM 2-Deoxy-D-Glucose and 10 mM NaN_3_ for ATP depletion (-ATP). Thereafter, cells were treated with hPep3/AF568-SSO complexes at 200 nM final concentration for 4 h in the presence of the inhibitors. Cells were washed twice with cold DPBS containing 100 µg/mL of heparin and detached with 0.05% trypsin-EDTA. The cell pellet was further washed with cold DPBS, and thereafter lysed in 0.1% Triton X-100. The endocytosis inhibition was evaluated by fluorescence spectroscopy and evaluated over hPep3/AF568-SSO treatments without inhibitors.

### 2.13. Animal Study

20-g female NMRI mice were subjected to intravenous injection with the formulations through the tails to compare the naked Cy5-SSO and hPep3/Cy5-SSO complexes in vivo at a total of 50 µg of Cy5-SSO at MR10 (3 animals per group). After 24 h, the animals were sacrificed, and organs harvested and imaged with fluorescence analysis using IVIS Spectrum (Perkin Elmer, Waltham, MA, USA). Data were processed by IVIS software (Living Image Software). Adobe Photoshop CS4 and Adobe Illustrator were used to crop out and align the organ images. The in vivo experiments were approved by the Swedish local laboratory animal research ethics committee (approval no. S4-16, approved in 5 January 2020) and all experiments were performed in accordance with relevant guidelines and regulations.

### 2.14. Statistical Analysis

Data are presented as mean with a standard error of the (mean ± SEM) of at least three independent experiments. Significant differences were evaluated by analysis of variance (ANOVA) with Dunnetts’s multiple comparison test, Bonferroni′s multiple comparison test or unpaired *t* test (GraphPad Prism 8 or 9; GraphPad Software, Inc., San Diego, CA, USA). In all cases, differences with *p* < 0.05 were deemed to be significant (* *p* < 0.05, ** *p* < 0.01, *** *p* < 0.001, and **** *p* < 0.0001).

## 3. Results

### 3.1. Design and Synthesis of Peptides

It is widely known that introduction of hydrophobic modifications into CPPs can improve the ability of CPPs to formulate highly stable nanoparticles via non-covalent complexation with oligonucleotides. In the current study we took inspiration from the chemical approach used in peptide stapling by ruthenium-catalyzed ring-closing metathesis (RCM). In this method different α,α-disubstituted alkenyl-alanine moieties with various lengths/hydrophobicities are introduced into the peptide sequence to provide rigidity into the peptide secondary structure via covalent bridging of the alkenyl moieties. Here, we repurposed hydrocarbon-modified amino acids used in RCM to orthogonally introduce different amounts of hydrophobicity into the peptide sequence. The rationale behind the design of the used peptide sequence was to create an almost ideal alpha-helical amphipathic peptide structure, where the charged Lys-residues are distinctly separated from the hydrophobic Leu-residues by neutral alanine residues. The residues with the hydrophobic alkenyl-alanines were placed in the middle of the hydrophobic side of the helix which increases the amphipathic moment of the helix. As can be seen from the UPLC data at constant gradient in Figure S1, the hydrophobicity increases in the following order hPep1 < hPep2 < hPep3 with 13, 16 and 24 total carbons over all added alkenyl residues, respectively. All peptides carry 6 positive charges from which 5 come from side chains of lysines and one comes from the N-terminus Table 1.

### 3.2. Physicochemical Characterization of hPep Peptide/SSO Complexes

**Figure 1:**
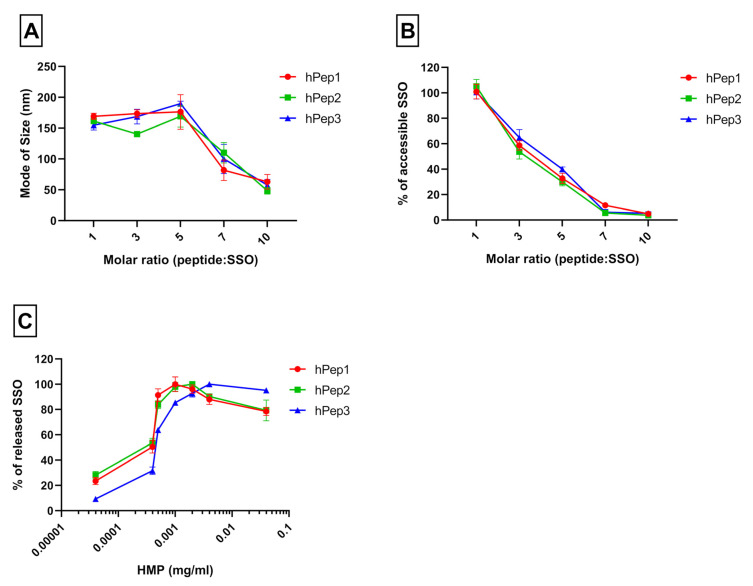
Physicochemical characterization and stability of hPep/SSO nanoparticles. (**A**) Size of the peptide/SSO nanoparticles formulated over a range of peptide-to-SSO molar ratios evaluated by Nanoparticle Tracking Analysis (NTA). (**B**) The encapsulation efficiency of the peptide/SSO nanoparticles was measured by SYBR Gold exclusion assay. (**C**) The same assay was used to evaluate the stability of the complexes, challenged by the presence of a competitive polyanion, hexametaphosphate (HMP), to release the SSO. Values indicate the mean of three independent experiments measured in triplicates (mean ± SEM).

One way to vectorize nucleic acid/ONs with peptides is by a non-covalent nanoparticle formation strategy. This approach relies on the ability of the cationic peptides to associate and form nano-complexes/-particles with negatively charged ONs. The process is primarily driven by the electrostatic interaction, but both hydrophobic interactions and hydrogen bonding also play an important role in the formation of stable nanoparticles [12]. To generate peptide/SSO nanoparticle formulations, peptides are usually used at different peptide/SSO molar ratios (MRs) to find optimal conditions and it is known that this parameter influences the rate of encapsulation of SSOs as well as the physicochemical properties of the complexes. To study the ability of the hPep peptides to form peptide/SSO nano-complexes, the SSO was formulated with hPep peptides at peptide/SSO MRs from 1 to 10, and nanoparticle formation was evaluated by using a Nanoparticles Tracking Analysis (NTA) and SYBR Gold exclusion assay. The NTA showed that all hPep peptides were able to associate with SSOs and form nanoparticles with a size of around 150–200 nm between molar ratios 1 and 5. Beyond that, size started to decrease to around 100 nm at MR7 and 50 nm at MR10 (Figure 1A). This effect was in line with SYBR Gold exclusion assay, where MR-dependent encapsulation of SSO into nanoparticles was observed with almost no complexation at MR1 to full complexation with over 90% encapsulation efficiency (Figure 1B) at MR7 and MR10. Altogether, we did not observe any major difference in the complex formation nor size when we compared hPep peptide derivatives with different hydrophobicities. Although differences in the size and encapsulation characteristics could not be observed between peptide derivatives, it is known that hydrophobicity plays a key role in the stability of the peptide/ON nanoparticles. To test the relative stability of the various hPep peptide/SSO complexes, we used a competitive polyanion, hexametaphosphate (HMP), to dissociate and release the SSO from the nanoparticles. When nanoparticles were treated with an increasing concentration of HMP, a clear correlation between the hydrophobicity of the peptide and the stability of the complexes could be observed, where more hydrophobic peptides formed nanoparticles with higher stability (see Figure 1C).

### 3.3. hPep/SSO Nanoparticles Induce Effective Splice-Switching Activity In Vitro

**Figure 2:**
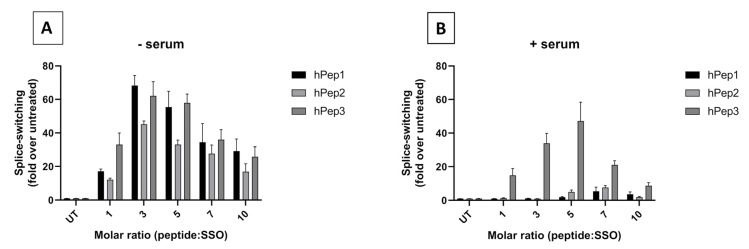
Delivery efficacy and optimization of the formulation conditions for hPep/SSO nanoparticles in the HeLa 705 reporter cell line. Cells were treated with peptide/SSO nanoparticles formulated at different MRs (**A**) in serum-free media using 100 nM SSO or (**B**) serum-containing media using 200 nM SSO for 24 h. Thereafter, luciferase expression was measured by luminometric analysis. Splice-switching activity is presented as a fold-increase in luciferase activity over untreated cells. Values indicate the mean of at least three independent experiments measured in triplicates (mean ± SEM).

After confirming the ability of the peptides to form nanoparticles at different MRs, we next wanted to study if the hPep peptides are also capable of effective delivery of the SSOs to the cells. For this we used an HeLa pLuc705 in vitro splice-switching model developed by Kole and colleagues [14]. In this model, HeLa cells are stably expressing luciferase genes interrupted by mutated beta-globin intron 2 carrying a cryptic splice site. This prevents the removal of the intron and results in the production of aberrantly spliced non-functional luciferase. Sterical masking of this aberrant splice site with SSO restores the splicing and the production of functional luciferase. This makes it an excellent positive read-out system for evaluating functionality and efficacy of new SSO therapeutics in the nucleus of cells. To evaluate the delivery capacity of hPep peptides for SSOs, HeLa pLuc705 cells (hereafter referred to as HeLa 705) were treated with hPep/SSO nanoparticles formed over a range of MRs in the serum-free transfection media. At these conditions, all hPep peptides induced high levels of splice-switching activity as measured by increased levels of luciferase expression (Figure 2A). Strikingly, all hPep peptides were able to induce high splice-switching activity, with the activity being most pronounced in the case of hPep1 and hPep3 formulated at MR3 and MR5. At the same time, hPep2 was also able to induce high levels of splice-switching activity; however, it was slightly less active than the hPep1 and hPep3 in the serum-free conditions (Figure 2A). The delivery capacity of various drug delivery vectors is known to be highly sensitive to the presence of serum proteins in the transfection media. To better understand how the presence of serum would affect the hPep-mediated delivery of SSO, we next carried out the transfection in serum-containing media. Interestingly, in the presence of serum, hPep1 and hPep2 were much less effective and only low level of activity was seen at the MR7 for both peptide derivatives (Figure 2B). In comparison, the most hydrophobic hPep3 peptide was significantly more resistant to the presence of serum and was able to retain its activity and induce high levels of splice-switching (Figure 2B). The most optimal conditions for achieving the highest level of splice-switching efficiency with hPep3 were observed when the peptide/SSO nanoparticles were formed at MR5. Based on this, MR5 was chosen for the next set of experiments.

### 3.4. hPep/SSO Nanoparticles Mediate Dose-Dependent Splice-Switching Activity

**Figure 3:**
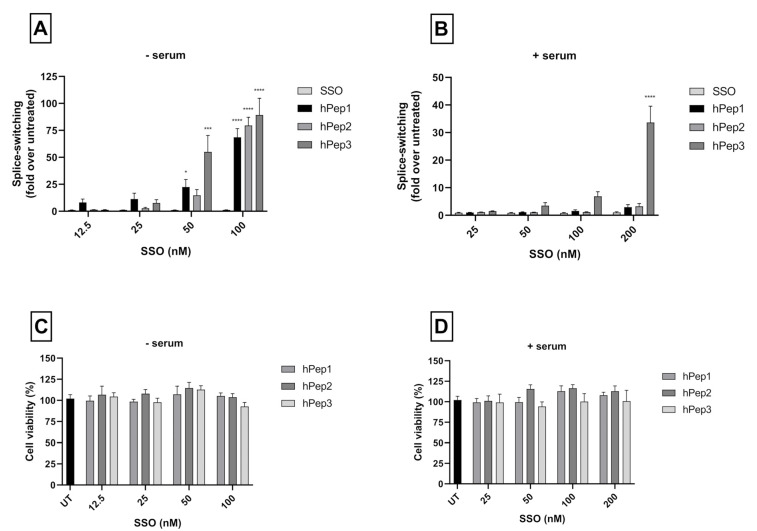
Dose-dependent splice-switching activity and toxicity evaluation of hPep/SSO nanoparticles in HeLa 705 reporter cell line. The efficacy of the complexes was examined via dose–response experiments in serum-free (**A**) and serum-containing transfection conditions (**B**). Splice-correction results are shown as fold-increase in luciferase activity over levels that were obtained from untreated cells. In a similar setup the cytotoxicity of the complexes was examined by WST-1 in serum-free (**C**) and serum-containing media (**D**). The cytotoxicity data are presented as percentage of untreated cells. The presented values are the mean of at least three independent experiments conducted in duplicates (mean ± SEM). *p*-values were determined by one-way ANOVA with Dunnett′s multiple comparison test (* *p* < 0.05, *** *p* < 0.001, **** *p* < 0.0001).

After demonstrating the ability of hPep peptides to mediate effective delivery of SSOs, we next wanted to explore the pharmacological activity profile of the hPep/SSO nanoparticles in more detail. To get a better insight into this, we carried out dose-response experiments to evaluate both the splice-switching activity and potential adverse effects of the transfections on cell viability. For this, HeLa 705 cells were treated with hPep/SSO nanoparticles containing different hPep peptide formulations over a range of SSO concentrations for 24 h both in serum-free and serum-containing media. In serum-free conditions, all hPep peptides demonstrated an ability to induce significant splice-switching activity in a dose-dependent manner (Figure 3A).

When the transfections were carried out in the presence of serum proteins, only the most hydrophobic peptide, hPep3, was able to retain activity and induce significant splice-switching activity in a dose-dependent manner (35-fold increase at 200 nM SSO), in line with the effects seen in the molar ratio titration experiment above. At the same time, formulations with hPep1 and hPep2 were only able to induce very low levels of splice-switching activity at the highest 200 nM SSO concentration, while control treatments with naked SSOs were completely inactive (Figure 3B). Furthermore, all hPep formulations with irrelevant control SSO used in both treatment conditions were unable to induce any splice-switching activity, indicating both high specificity and lack of off-target effects related to the hPep/SSO treatments (data not shown).

Another important aspect to consider when developing effective DDSs is that it should mediate delivery in a non-toxic manner. First line toxicity can be evaluated by using different cell proliferation assays. To assess whether the hPep3 superiority in transfection activity is related to its higher tolerability by the cells we evaluated the toxicity to the cells by WST-1 cell proliferation assay. By measuring the metabolic activity of the cells with WST-1 we were able to conclude that none of the hPep formulations had adverse effects on cell viability at any of the tested concentrations in both serum-free and -containing media (Figure 3C,D). 

**Figure 4:**
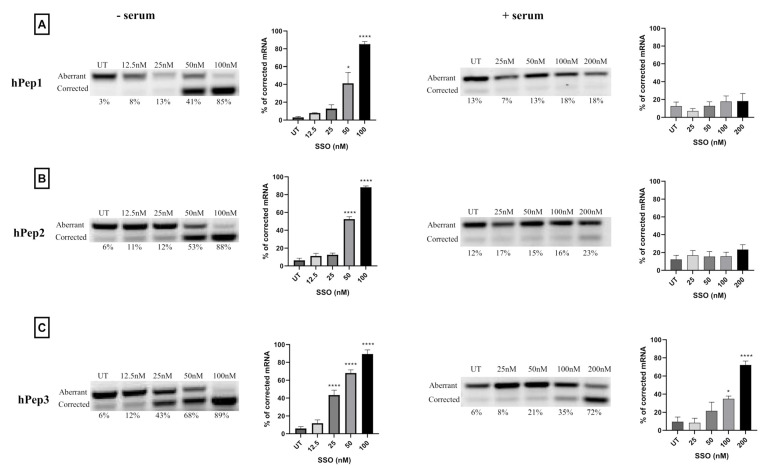
Splice-switching activity evaluation on luciferase mRNA level in HeLa 705 reporter cell line. Cells were transfected with increasing concentrations of hPep/SSO formulations. Cells were incubated with hPep/SSO nanoparticles for 24 h in both serum-free media (panels on the left) and serum-containing media (panels on the right) with (**A**) representing hPep1, (**B**) hPep2 and (**C**) hPep3. Splice-switching activity on the mRNA level was analyzed with RT-PCR. Products were separated by gel electrophoresis and correction values were derived from densitometric analysis. Representative gel for every condition is displayed (upper band = uncorrected mRNA, lower band = corrected mRNA). Bar graphs represent the mean % of mRNA correction derived from at least three independent experiments (mean ± SEM). *p*-values were determined by one-way ANOVA using Dunnett′s multiple comparison test (* *p* < 0.05, **** *p* < 0.0001).

After confirming that the hPep/SSO nanoparticles were able to mediate dose-dependent splice-switching activity on the protein level in the luciferase induction assays, we next sought to investigate the conversion of the aberrant-to-corrected transcript on the luciferase mRNA level. Our experiments showed that different hPep/SSO formulations were able to mediate highly effective dose-dependent splice-switching activity on the mRNA level as measured by RT-PCR (Figure 4). The data indicated that in serum-free conditions half-maximal effective concentration (EC50), i.e., the level where 50% mRNA is corrected, was 52 nM for hPep1, 49 nM for hPep2 and 31 nM for hPep3, respectively (Figure S2). At 100 nM SSO concentration, all peptides could produce close to maximal level of corrected luciferase mRNA transcript at around 75–90%. In line with the results above, evaluation of the splice-switching activity in the presence of serum proteins showed that hPep3 was able to correct approximately 70% of the aberrant mRNA at the 200 nM SSO concentration (Figure 4) and displayed an EC50 value of 104 nM (data not shown). As compared to the serum-free conditions, hPep3 correction levels in the presence of serum were on par with the levels achieved in serum-free conditions at 100 nM SSO (Figure 4). In line with the data from the molar ratio and dose-titration experiments above (Figure 2 and Figure 3), both hPep1 and hPep2 had very limited activity in the serum-containing transfection conditions (Figure 4) and accordingly EC50 values could not be calculated.

### 3.5. Quantitative Uptake and Intracellular Localization of Peptide/SSO Nanoparticles

**Figure 5:**
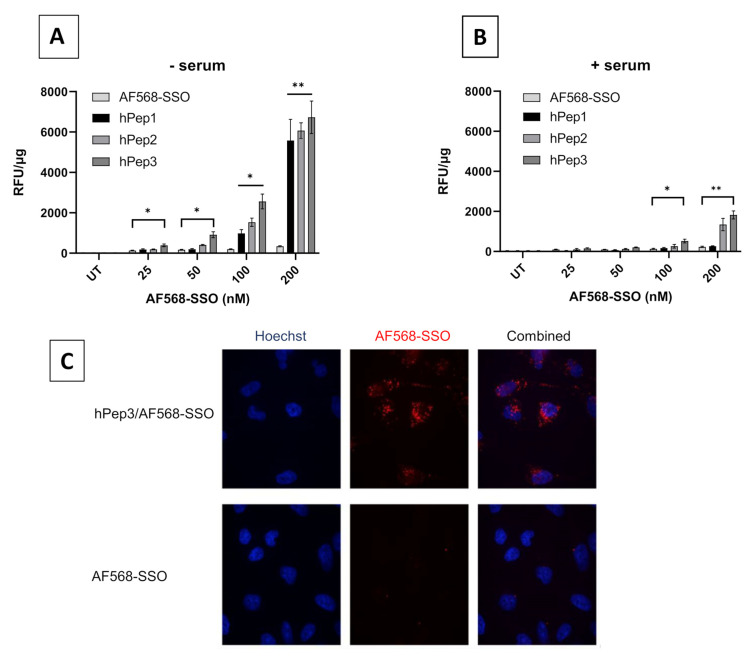
Quantitative uptake and intracellular localization of hPep/SSO nanoparticles in HeLa 705 cells. For quantitative uptake studies, cells were treated for 4 h over a range of concentrations with AF568-SSO complexes formulated both in (**A**) serum-free and (**B**) serum-containing media and the uptake was analyzed by fluorescence spectroscopic analysis. (**C**) Cellular localization of hPep3/AF568-SSO nanoparticles (red) was followed by live cell imaging with confocal microscopy 4 h after the treatments. Then the nuclei were stained with Hoechst (blue). The presented values are the mean of at least three independent experiments conducted in duplicates (mean ± SEM). *p*-values were determined by one-way ANOVA with Dunnett′s multiple comparison test (* *p* < 0.05, ** *p* < 0.01).

In order to better understand the role of different alkenyl modifications in the cellular uptake of various hPep/SSO nanoparticles, we next aimed to study their quantitative uptake into the cells with fluorescently labelled nanoparticles. For that, hPep/SSO nanoparticles were formed with an Alexa Fluor 568-labelled SSO (AF568-SSO) and the HeLa 705 cells were treated at different concentrations in serum-free and serum-containing media and uptake was then measured by fluorescence spectroscopy. The data indicated that at higher concentrations in serum-free conditions, all the peptides were able to significantly increase the uptake of AF568-SSOs in a dose-dependent manner as compared to the control treatments with naked AF568-SSO. At the same time, hPep3 was clearly most potent in facilitating the uptake of the AF568-SSO at the lower concentration and was overall most effective in serum-free transfection conditions. Next, we also analyzed the uptake in serum-containing conditions and here the more hydrophobic peptides, hPep2 and hPep3, were able to retain their delivery efficacy, while hPep3 also here was clearly the most effective. At the same time hPep1/AF568-SSO nanoparticles readily lost their ability to mediate effective uptake of SSO in the presence of serum proteins. It could also be observed that the uptake for all the hPep peptides was much lower in the presence of serum proteins as compared to the serum-free conditions. Overall, these data indicate that the peptides with increasing number of alkenyl modifications and increased hydrophobicity provided higher levels of cellular uptake for the AF568-SSO (Figure 5A,B).

After confirming that the hPep/SSO nanoparticles were taken up by the cells we next wanted to study the intracellular distribution and localization of the hPep/SSO nanoparticles and chose to carry out the next experiments with the most potent hPep derivative, the hPep3 peptide. To study the cellular distribution of the nanoparticles, we treated the HeLa 705 cells with hPep3/AF568-SSO nanoparticles and followed their uptake/distribution with confocal microscopy live cell imaging. As seen in Figure 5C, hPep3 nanoparticles can be visualized as a punctuated fluorescent staining inside cells, which is indicative of endosomal localization, while there seems to be very limited diffuse cytoplasmic stain detectable inside the cells. This indicates that hPep3/SSO nanoparticles are most likely taken up via endocytic transport processes. 

### 3.6. Uptake of the hPep/SSO Nanoparticles Is Driven by Endocytosis

**Figure 6:**
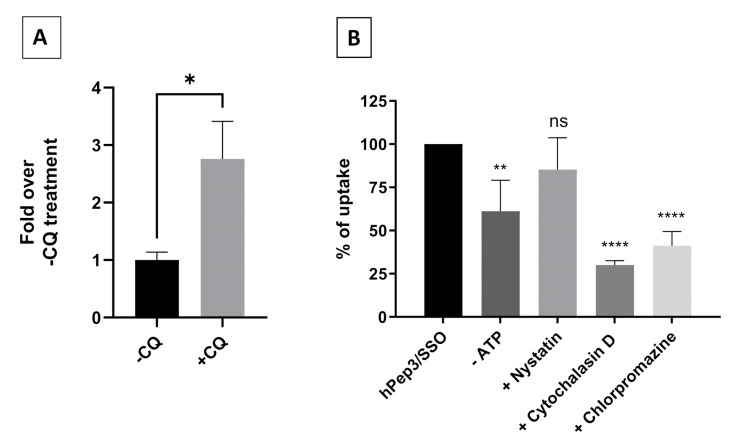
Cellular trafficking of hPep3/SSO nanoparticles in HeLa 705 cells. (**A**) Cells were treated with a 200 nM of hPep3/SSO nanoparticles for 4 h with (+CQ) or without (-CQ) chloroquine treatment and luminescence activity was measured 18 h later. The values are the mean of at least the three experiments (mean ± SEM). *p*-values were determined by the independent *t*-test (* *p* < 0.05). (**B**) Impact of endocytosis inhibitors and energy depletion to the uptake of hPep3/AF568-SSO complexes. For inhibition the following inhibitors were used: 2′-Deoxy-D-Glucose and NaN_3_ for ATP-depletion (-ATP); nystatin for caveolae-mediated endocytosis inhibition; and cytochalasin D for micropinocytosis; and chlorpromazine for clathrin-mediated endocytosis inhibition. The values are the mean results of at least three independent experiments (mean ± SEM). *p*-values were determined by the one-way ANOVA with Dunnett’s multiple comparison test (ns—non-significant, * *p* < 0.05, ** *p* < 0.01 and **** *p* < 0.0001).

CPPs are usually considered to use various paths of endocytosis to gain access to the inside of cells, particularly when bound to cargo molecules. Together with our findings from live cell imaging studies above, these data indicate that the hPep/SSO nanoparticles are most probably entrapped in the endocytic vesicles in the endo-lysosomal system. To confirm if the hPep3/SSO complexes are entrapped in the endosomes we next chose to use endosomolytic chloroquine co-treatment to release the material from the endosomal system. As seen in Figure 6A, chloroquine co-treatment significantly increased the splice-switching activity of the hPep3/SSO nanoparticles (up to 2.5-fold) over the control treatment, supporting the notion that these nanoparticles were taken up by the endocytic route and indicating that a considerable portion of the material remains entrapped in the endolysosomal system (Figure 6A).

Various endocytic pathways have been shown to be involved in the cellular uptake of CPPs [15]. To further corroborate the involvement of endocytosis and also shed light on which specific endocytic pathway could be involved in the uptake of hPep3/SSO nanoparticles, we next chose to study the hPep3/SSO nanoparticles in the presence of pharmacological inhibitors of the main endocytic pathways. To inhibit the various endocytic pathways, we used nystatin for caveolae-mediated endocytosis (CAE), cytochalasin D for macropinocytosis (MP), chlorpromazine for clathrin-mediated endocytosis (CLE) and NaN3 and 2′-Deoxy-D-Glucose for ATP-depletion (-ATP). As seen in Figure 6B, uptake of hPep3/SSO nanoparticles follows energy-dependent pathway as ATP-depletion decreased the uptake. Moreover, cytochalasin D and chlorpromazine significantly decreased the uptake indicating the involvement of both macropinocytosis and clathrin-mediated endocytosis in the cellular uptake of the hPep3/SSO nanoparticles. At the same time, caveolae-mediated endocytosis had negligible effect on the uptake of the hPep3/SSO nanoparticles as treatments with nystatin had insignificant effect on the uptake (Figure 6B).

### 3.7. In Vivo Biodistribution of hPep/SSO Nanoparticles Is Dominated by Enhanced Accumulation in Liver, Spleen and Kidneys

**Figure 7:**
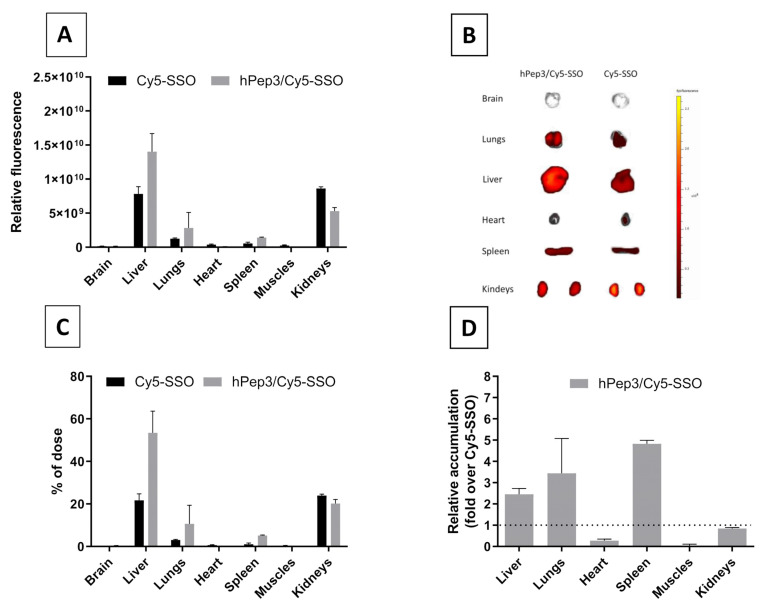
Biodistribution of hPep3/SSO nanoparticle formulation in vivo. (**A**) Biodistribution of hPep3/SSO nanoparticles in mice was evaluated upon intravenous injection of hPep3/Cy5-SSO nanoparticles or naked Cy5-SSO (at 2.5 mg/kg of SSO). 24 h after the injection, the tissues were harvested and the biodistribution evaluated by quantifying the total fluorescence with IVIS in vivo imager. (**B**) Representative IVIS images of the biodistribution of hPep3/Cy5-SSO and naked Cy5-SSO in different organs. (**C**) Percentage of the fluorescence signal from the total signal in different organs. (**D**) Relative increase in accumulation of the hPep3/Cy5-SSO nanoparticles compared to naked Cy5-SSO in different organs (presented as fold-increase over Cy5-SSO signal). *N* = 3.

Despite recent advances in oligonucleotide chemistry to improve in vivo pharmacokinetics, SSOs lack the ability to achieve efficient body wide distribution. After confirming the efficiency and safety of the hPep3/SSO nanoparticles in cell culture, we next sought to determine if hPep3 can be used to improve delivery and biodistribution profile of SSOs in vivo. Upon systemic i.v. delivery in mice, hPep3/Cy5-SSO nanoparticles displayed a broad systemic in vivo biodistribution, while the free Cy5-SSO primarily accumulated in the liver and kidneys (Figure 7A,B). As compared to the free Cy5-SSO, hPep3/Cy5-SSO nanoparticles increased the Cy5-SSO accumulation in liver, lungs and spleen (Figure 7C) by up to 2.5, 3.5 and 5-fold, respectively (Figure 7D). Overall, treatments with hPep3/Cy5-SSO nanoparticles were well tolerated and were able to improve the biodistribution profile of SSOs in vivo.

## 4. Discussion

The numerous ON-based methods for gene function modulation all face the same challenge of effective delivery to target cells. This inherent limitation is shared by antisense ONs and SSOs, implying that improving the delivery of this class of therapeutic molecules is very important for the wider clinical translation of ON therapeutics. Broader interest in the development of ON-based therapies has also intensified efforts in finding more effective and safe delivery vectors for both in vitro and in vivo applications. The use of CPPs to enhance cellular uptake and bioactivity of different types of ONs represents one approach that offers great promise for improving the bioavailability of ON-based compounds. Different chemically modified ONs, such as locked nucleic acids (LNAs), peptide nucleic acids (PNAs), and 2′-O-Methyl RNA are being used to modify aberrant splicing patterns. SSOs have been successfully applied to modulate splicing patterns in the context of various splicing disorders, such as for spinal muscular atrophy and Duchenne muscular dystrophy [16,17,18]. These findings emphasize the therapeutic potential of SSOs in human diseases; however, further development is necessary to enhance efficiency and systemic delivery of SSOs. To enhance delivery, SSOs are usually complexed with positively charged DDSs to form nanoparticles [19]. CPPs are one such versatile class of nucleic acid DDSs, which are able to formulate therapeutic oligonucleotides of different sizes, including SSOs, into nanoparticles. However, many challenges remain, such as low colloidal stability and poor endosomal release of the nanoparticles. 

A common way to increase the cellular uptake of CPP/SSO nanoparticles is by increasing the hydrophobic interactions with the membrane via lipidation of the CPPs [13,20,21]. This is usually achieved by adding hydrocarbon chains with varying length to the N-terminus of the CPPs. While this approach comes with many advantages it can also bring unwanted side-effects such as increased hemolytic activity [12]. In the current study, we aimed to find an alternative chemical approach for CPP lipidation and designed new chemically modified CPPs called hPep′s using orthogonal hydrocarbon modifications with alkenyl-alanines to introduce hydrophobicity into the peptide backbone. The advantage of this approach is that the alkenyl-alanines can be incorporated into the peptide backbone at different locations during the automated synthesis stage. Therefore, it makes this approach very versatile and fine-tunable as it gives the opportunity to choose the number and length of the hydrocarbon modifications based on the application. In fact, we find that complexes formulated with hPep3 peptide with three octenyl modifications (24 added carbons) have higher uptake than hPep1 and hPep2 with 13 and 16 carbons (Figure 5), while all of them form almost identically sized nanoparticles, in the 50–200 nm range. This is similar to our previous findings with PepFect/SSO nanoparticles, where it was found that at least 12 carbon atoms were required for the complexes to start associating with the cell membrane, and the higher the number of carbon atoms the more effectively the complexes associated with the cells [12].

In order to determine the optimal peptide/SSO molar ratio, we performed screening of different MRs in the HeLa 705 cell line. We found that splice-switching activity was the highest between MRs 3 and 7 in both serum-free and serum-containing conditions (Figure 2). Hence, we chose MR5 for subsequent detailed studies. Another aspect supporting choosing the MR5 is that it has been shown for different cationic DDSs that complexation of ONs with excessive amounts of delivery vectors leads to unwanted toxicity caused by the unbound free fraction of DDS due to its membrane active properties [12,22,23]. 

More detailed cell culture studies with hPep/SSO complexes showed that out of all the tested peptides, only the most hydrophobic hPep3 was able to induce significant splice-switching activity both at the luciferase as well as the mRNA (EC50 = 104 nM) level at serum-containing conditions. To our surprise, these results are very well in line with our previous findings with N-terminal fatty acid analogs of PepFect14, where we showed, by varying the hydrocarbon chain length, that by introducing ≥12 carbons, splice-correction activity of the SSO complexes increased linearly with each added carbon [12]. We hypothesize that the superior activity of the hPep3 could be due to the higher stability, as hPep3 nanoparticles were much more stable towards competitive polyanion treatment than the rest of the peptides (Figure 1C), correlating well with other similar findings on the importance of hydrophobic interactions for the stability of CPP/nucleic acid complexes [11,24]. Furthermore, none of the tested peptides showed any adverse effect on the cell viability, even at the 200 nM SSO treatment concentration. Together these data imply that introduction of orthogonal hydrocarbon modifications into the peptide to increase its hydrophobicity can be used to generate a highly efficient and well-tolerated drug delivery system for SSOs in cell cultures.

It is widely recognized that nanoparticle-based DDSs mainly enter cells via endocytosis and end up in endocytic vesicles. When endosomes mature into lysosomes the pH in these vesicles drops, which activates lysosomal enzymes that will degrade the contents inside the vesicles. Therefore, DDSs have to escape the endosomes with their payload before they get degraded. Endosomal entrapment is considered one of the major bottlenecks for all DDSs. Similarly to other DDSs, we found that the hPep3/SSO nanoparticles to a high extent get entrapped in the endosomes, as treatment with endosomolytic agent chloroquine could be used to release the endosome-bound material (Figure 6A). When we delved more into the cellular uptake mechanisms with specific pharmacological inhibitors it was clear that hPep3/SSO NPs enter cells via energy-dependent pathways, mainly, by macropinocytosis and clathrin-mediated endocytosis. The use of several endocytic pathways has also been reported for other peptide-based ON delivery systems. For example, PepFect14/SSO NPs have been shown to enter cells via macropinocytosis and caveolin-dependent endocytosis while involvement of scavenger receptors during these processes has been documented in several studies [25,26,27].

After confirming the safety and efficacy of hPep3/SSO NPs in vitro we went on to study its delivery capabilities in vivo. Biodistribution studies in mice with the fluorescently labelled hPep3/SSO nanoparticles indicated that hPep3 could increase the accumulation of SSOs in liver, lungs and spleen by up to several folds as compared to the treatments with free SSO (Figure 7D). Furthermore, the i.v.-administered complexes were very well tolerated by the animals. 

## 5. Conclusions

Taken together, we demonstrate an alternative chemical approach for the orthogonal introduction of hydrophobic modifications into CPPs by repurposing the alkenyl-alanines used in peptide stapling and show how this approach can be successfully applied to develop efficient and well-tolerated CPP-based delivery systems for the nanoformulation of therapeutic SSOs.

## Figures and Tables

**Table 1:** The structures and properties of the peptides used in the study.

Name	Sequence	Charge	MW (g/mol)	RT (min)
hPep1	H-LAKLAKA(R8)AKLLKA(S5)AKAL-NH2	+6	2040.4	9.8
hPep2	H-LAKLAKA(R8)AKLLKA(S8)AKAL-NH2	+6	2084.4	10.7
hPep3	H-L(R8)KLAKA(R8)AKLLKA(R8)AKAL-NH2	+6	2193.9	13.2

(R8) = (R)-2-(7-octenyl)alanine; (S8) = (S)-2-(7-octenyl)alanine; (S5) = (S)-2-(4-pentenyl)alanine; RT—retention time.

## Data Availability

Not applicable.

## Supplementary Materials

The following are available online at https://www.mdpi.com/article/10.3390/biomedicines9081046/s1, Figure S1 shows UPLC chromatograms of hPep peptides. Figure S2 shows the half-maximal effective concentrations for hPep/SSO complexes in HeLa 705 cells on mRNA level.
