# Supplementary material for: Novel Orthogonally Hydrocarbon-Modified Cell-Penetrating Peptide Nanoparticles Mediate Efficient Delivery of Splice-Switching Antisense Oligonucleotides In Vitro and In Vivo
Source: Biomedicines. 2021 Aug 19;9(8):1046. doi: 10.3390/biomedicines9081046 (PMC8392223; doi:10.3390/biomedicines9081046)
Supplement: Supplementary file 1 [file biomedicines-09-01046-s001.zip › biomedicines-1323472-supplementary.pdf]

Supplementary Information

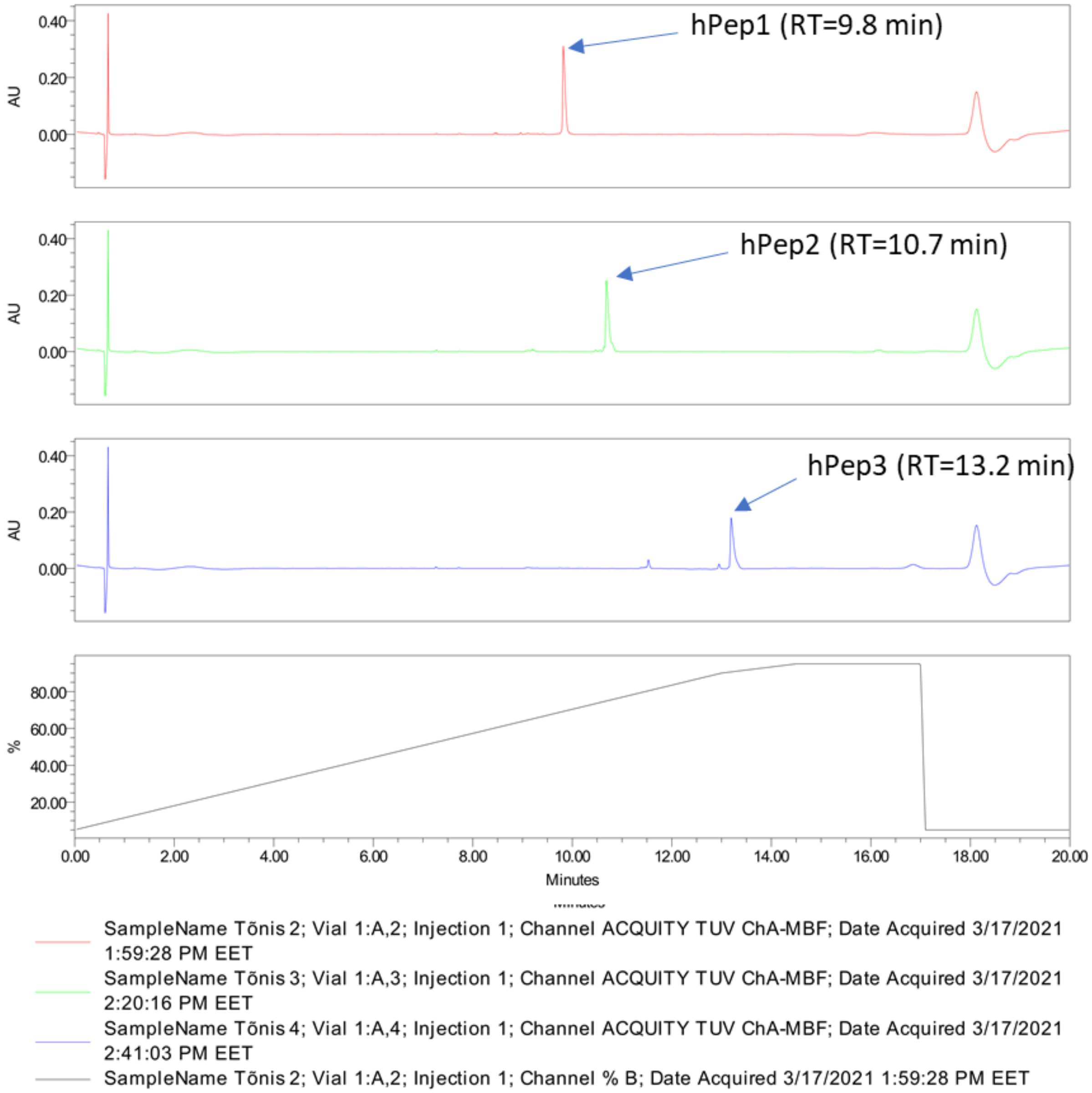

Supplementary figure S1. UPLC chromatograms of hPep peptides. In order to compare the hydrophobicities of the peptides we used RP-UPLC (Waters, Milford, MA) with AcN/water (0,1% TFA) gradient running from 5-90% in 13 min using a Acquity UPLC BEH130 C18 column (1.7um 2.1 x 100mm).

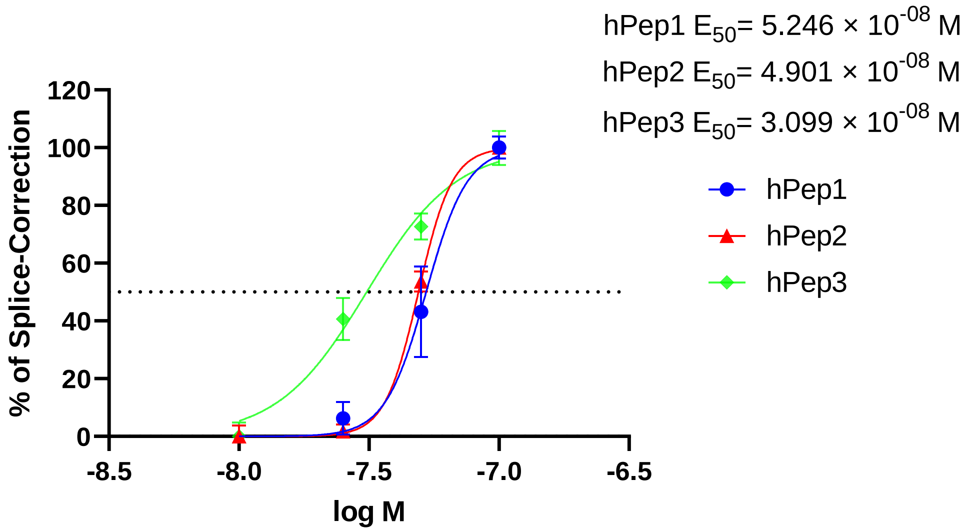

Supplementary figure S2. Half-maximal effective concentrations for hPep/SSO complexes in HeLa 705 cells on mRNA level. EC_{50} values were calculated from RT-PCR data with GraphPad Prism software from the 24 h treatment in serum-free conditions.
